# Refractory *Candidozyma* (*Candida*) *auris*-associated central nervous system infection in a postoperative neurosurgical patient

**DOI:** 10.1128/asmcr.00008-26

**Published:** 2026-04-15

**Authors:** Yuan Chao Xue, Khadija Thomas, Noor Zaidan, Ping Ren

**Affiliations:** 1 Department of Pathology, University of Texas Medical Branch 12338, Galveston, Texas, USA; 2 Department of Pharmacy, University of Texas Medical Branch 12338, Galveston, Texas, USA; Pattern Bioscience, Austin, Texas, USA

**Keywords:** *Candidozyma *(*Candida*)* auris*, central nervous system infection, antifungal resistance, therapeutic management

## Abstract

**Background:**

*Candidozyma* (*Candida*) *auris* has emerged as a global health threat because of its ability to persist on skin and environmental surfaces and its frequent resistance to multiple antifungal agents. We describe a rare but clinically significant case of *C. auris*-associated central nervous system (CNS) infection in a postoperative neurosurgical patient.

**Case Summary:**

A 60-year-old woman developed a postoperative cerebrospinal fluid (CSF) leak following intradural lumbar tumor resection and was readmitted with headaches, neck pain, and nausea. CSF and wound cultures grew *C. auris*, while blood cultures remained negative, suggesting localized CNS involvement. Combination antifungal therapy with liposomal amphotericin B, flucytosine, and micafungin, later supplemented with posaconazole, was initiated but was complicated by significant toxicities requiring dose adjustments and stepwise discontinuation. Antifungal susceptibility testing demonstrated reduced susceptibility to amphotericin B, and *C. auris* was repeatedly isolated from CSF despite therapy. Given persistent recovery of the organism, the clinical team initiated the US Food and Drug Administration Single Patient IND process to obtain fosmanogepix, an investigational antifungal agent, although the request was ultimately discontinued as the patient clinically stabilized. She was discharged on long-term oral voriconazole for antifungal suppression because of persistent CSF isolation of *C. auris*, multidrug resistance, and limited CNS penetration of previously administered antifungals.

**Conclusion:**

This case highlights diagnostic uncertainty, therapeutic challenges, and antifungal decision-making complexities following isolation of *C. auris* from CSF.

## INTRODUCTION

*Candidozyma* (*Candida*) *auris* is an emerging multidrug-resistant fungal pathogen that poses a growing global health threat because of its ability to persist on skin and environmental surfaces and cause healthcare-associated outbreaks. Since its first description in 2009, *C. auris* has been increasingly reported worldwide and is frequently associated with invasive infections and limited antifungal treatment options. Most reported cases involve bloodstream, wound, or urinary tract infections, whereas central nervous system (CNS) involvement remains rare and poorly described in the literature. Diagnosis of CNS infection due to *C. auris* is challenging because clinical findings may overlap with postoperative complications, and laboratory identification often requires specialized methods such as matrix-assisted laser desorption/ionization time-of-flight mass spectrometry (MALDI-TOF MS). We report a postoperative neurosurgical case with repeated isolation of *C. auris* from cerebrospinal fluid (CSF), highlighting the diagnostic uncertainty and therapeutic challenges associated with this uncommon presentation.

## CASE PRESENTATION

A 60-year-old female with diabetes and obesity, previously living independently in the community, initially presented with progressive gait instability for more than 2 months. Magnetic resonance imaging (MRI) revealed an intradural lumbar tumor. She was subsequently admitted for surgical management, and an L4-5 laminoplasty with L3-S1 laminectomies was performed for tumor resection. Eight days before surgery, thromboprophylaxis with enoxaparin had been initiated.

No additional oncologic treatment was planned following surgery. However, the patient presented to the emergency department (ED) 2 days postoperatively with complaints of CSF leakage and headache but was not admitted at that time. At a routine postoperative visit 10 days after surgery, a persistent CSF leak was noted at the incision site. Because her symptoms were improving and no clinical signs of infection were present, no additional follow-up was arranged.

**Fig 1:**
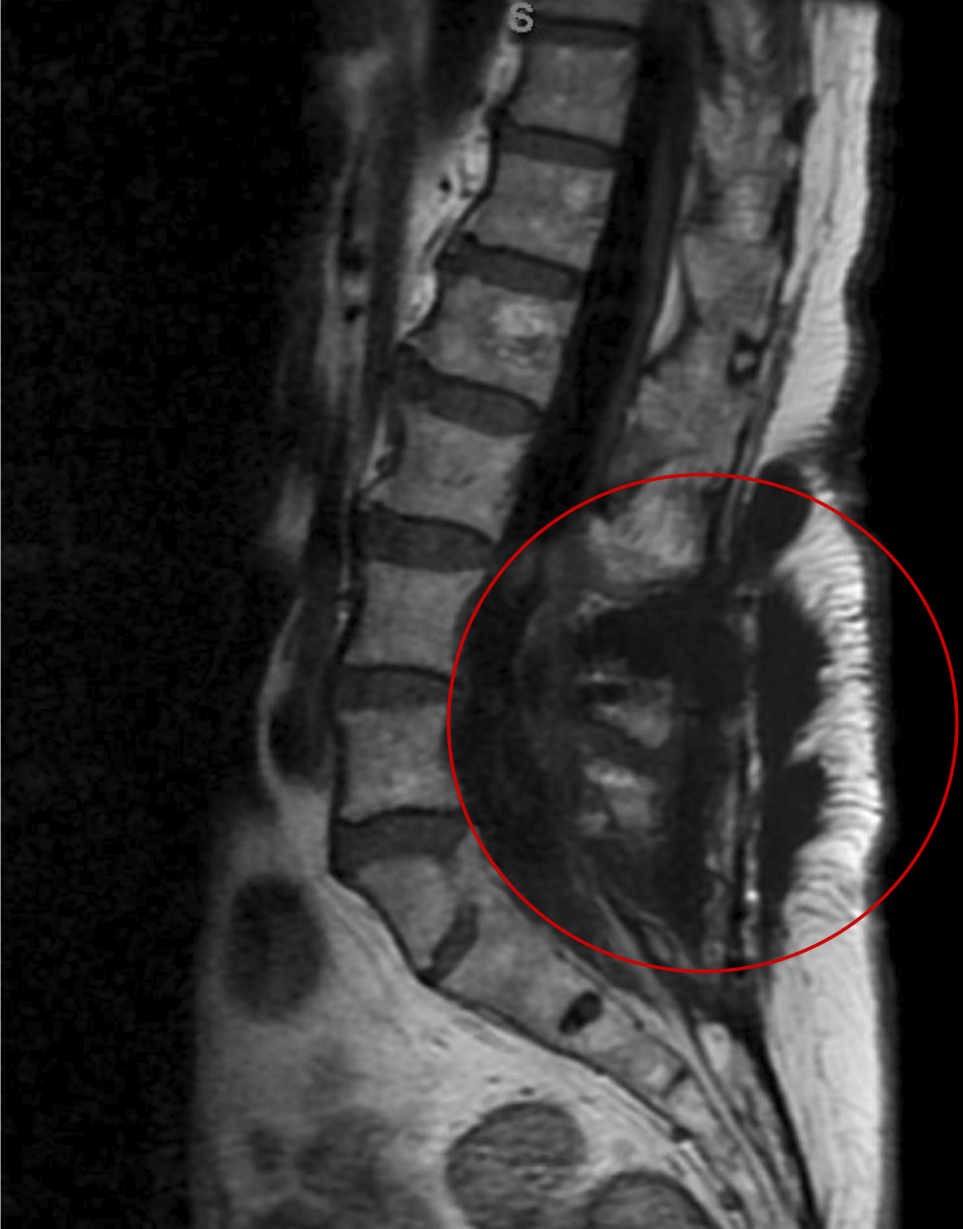
Magnetic resonance imaging of the lumbar spine on the day of admission. The red circle highlights the region of epidural fluid buildup.

**Fig 2:**
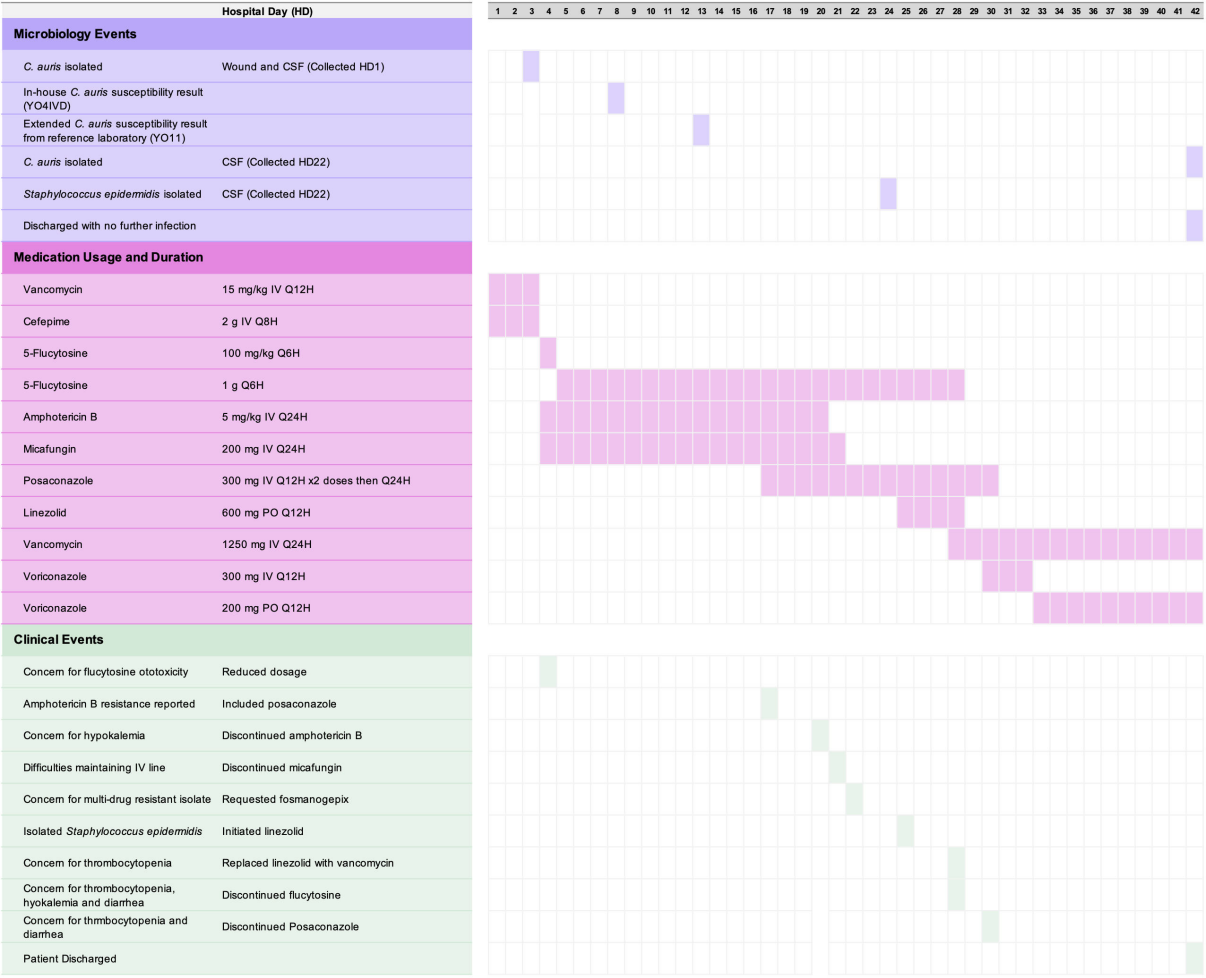
Medication and microbiology event summary.

On postoperative day 22, the patient was admitted to evaluate the headaches, neck pain, and nausea. MRI revealed postsurgical changes with a persistent complex, loculated fluid collection and interval enlargement of a collection in the laminectomy bed communicating with the overlying soft tissues (Fig. 1). These findings raised concern for a dural leak or pseudomeningocele, although infection could not be excluded. Medication and microbiology timelines are summarized in Fig. 2.

Given concern for a possible CNS infection, empiric therapy with vancomycin (15 mg/kg IV every 12 h) and cefepime (2 g IV every 8 h) was initiated. CSF from the lumbar wound leak, lumbar wound swabs, and blood cultures were obtained for microbiologic evaluation. CSF hematologic and chemistry analyses were not performed on the specimen. On hospital day 3 (HD3), both CSF and wound cultures grew *C. auris,* identified by MALDI-TOF MS, raising concern for localized *C. auris*-associated CNS involvement. Blood cultures remained negative.

Notably, a *C. auris* surveillance PCR of the axilla and groin performed 11 days earlier during the ED visit had been negative. This screening was conducted as part of routine ED surveillance, and no additional *C. auris* surveillance testing was subsequently performed.

Antifungal therapy was initiated with liposomal amphotericin B (5 mg/kg IV every 24 h), 5-Flucytosine (100 mg/kg [1,250 mg] every 6 h), and micafungin (200 mg IV daily) pending susceptibility results. Vancomycin and cefepime were discontinued after no bacterial pathogens were isolated. Flucytosine was reduced to 1 g every 6 h after 1 day due to ototoxicity.

On HD8, in-house antifungal susceptibility testing (using YO4IVD) results became available (Table 1). Additional susceptibility testing (using YO11) performed by the reference laboratory (ARUP, Salt Lake City, UT, USA) became available on HD13 demonstrated reduced susceptibility to amphotericin B (minimum inhibitory concentration [MIC] = 2 µg/mL) (Table 1), prompting the addition of posaconazole (300 mg IV every 12 h for two doses, followed by 300 mg every 24 h). Amphotericin B was continued despite the susceptibility profile because of the limited CNS penetration of alternative antifungal agents and the need to reduce the flucytosine dose due to ototoxicity.

**TABLE 1:** Susceptibility results of *Candidozyma (Candida) auris* isolates from CSF

Antifungal agent	*C. auris* from HD1 CSF		*C. auris* from HD22 CSF	*C. auris*
	Sensititre YeastOne YO4IVD MIC value (µg/mL) (reference range)	Sensititre YeastOne YO11^*b*^ MIC value (µg/mL) (reference range)	Sensititre YeastOne YO4IVD MIC value (µg/mL)	2024 CDC tentative MIC breakpoint (µg/mL)
Amphotericin B	NA^*a*^	2 (0.12–8)	NA	≥2
Anidulafungin	NA	0.12 (0.015–8)	NA	≥4
Caspofungin	0.25 (0.015–16)	0.12 (0.03–4)	0.5	≥2
Fluconazole	>128 (0.12–128)	≥128 (0.5–64)	128	≥32
Isavuconazole	NA	0.25 (0.008–4)	NA	NA
Itraconazole	0.25 (0.008–16)	0.25 (0.015–16)	0.12	NA
Micafungin	0.12 (0.008–16)	0.12 (0.008–8)	0.12	≥4
Posaconazole	NA	0.12 (0.008–8)	NA	NA
Rezafungin	0.12 (0.008–8)	0.12 (0.008–4)	0.12	NA
Voriconazole	1 (0.008–8)	1 (0.008–2)	0.5	NA
5-Flucytosine	0.12 (0.03–64)	NA	0.06	NA

^*a*^ NA, not available.

^*b*^ Performed by the reference laboratory, ARUP Laboratories (Salt Lake City, UT, USA).

Despite combination therapy, *C. auris* was again isolated on HD42 from a repeat CSF specimen obtained on HD22 with susceptibility patterns within two doubling dilutions of the initial isolate. Given the persistent recovery of the organism, the clinical team initiated the US Food and Drug Administration (FDA) Single Patient IND (SPIND) process to obtain fosmanogepix. However, this request was discontinued once the patient clinically stabilized and approached discharge.

During therapy, the patient developed hypokalemia, hypomagnesemia, difficulties maintaining IV access, thrombocytopenia, and diarrhea. These toxicities prompted stepwise discontinuation of antifungal agents: amphotericin B (HD20), micafungin (HD21), flucytosine (HD28), and posaconazole (HD30). Voriconazole (300 mg IV every 12 h) was started on HD29 and transitioned to 200 mg orally every 12 h on HD33. All adverse events had improved after a change in medication.

On HD 22, analysis of the second CSF obtained through lumbar drain demonstrated pleocytosis (1,151 cells/µL), and culture grew *Staphylococcus epidermidis* on HD24. *C. auris* was again isolated from the same CSF specimen on HD42. Linezolid (600 mg orally every 12 h) was initiated despite the lack of fever or clinical deterioration, but later changed to vancomycin (1,250 mg IV every 24 h) on HD27 due to thrombocytopenia. Vancomycin was discontinued on HD42.

The patient was discharged on HD42 afebrile, hemodynamically stable, and without recurrent headaches, neck pain, or visual symptoms. The surgical incision had healed completely, with no evidence of recurrent CSF leakage or infection. Long-term oral voriconazole was recommended for antifungal suppression because of persistent *C. auris* recovery from CSF, multidrug resistance, patient’s adverse responses to other antifungal agents, and the limited CNS penetration of previously administered antifungal agents. Close outpatient follow-up was arranged.

## DISCUSSION

*C. auris* was first identified in 2009 from the external ear canal of a patient in Japan, and its species name, “auris,” reflects its origin (1). Since then, it has emerged as a global health threat due to its persistence on skin and environmental surfaces, its ability to cause healthcare-associated outbreaks, and its high morbidity and mortality in invasive infections (1). Over the past decade, *C. auris* has been reported in more than 45 countries across six continents (1). Genomic analyses reveal that several clades emerged nearly simultaneously, indicating independent regional evolution rather than a single-source spread (2). In the United States, transmission is concentrated in high-acuity long-term care facilities where asymptomatic colonization of the nares, axilla, and groin serves as a major reservoir (1). Prevention relies on rapid and accurate detection, strict hand hygiene, contact precautions, and thorough environmental disinfection (3).

Morphologically, *C. auris* is a budding yeast that rarely forms pseudohyphae or true hyphae (4). Its ability to grow at high salinity (10% NaCl) and elevated temperatures (up to 42°C) contributes to its environmental resilience (4). Six major genomic clades: South Asian (Clade I), East Asian (Clade II), African (Clade III), South American (Clade IV), Iranian (Clade V), and Indo-Malaysian (Clade VI) are distinguished by characteristic single-nucleotide polymorphisms (2).

Diagnostic methods for *C. auris* include culture, phenotypic identification systems, MALDI-TOF MS, and molecular assays (5). Culture remains essential for susceptibility testing, although routine fungal culture cannot reliably distinguish *C. auris* from closely related yeasts based solely on colony morphology unless selective media are used. Selective media such as CHROMagar Candida Plus facilitate presumptive identification by producing light-blue colonies with a blue halo after 36–48 h at 37°C (5). Widely used phenotypic identification systems (e.g., VITEK 2 YST, API 20C, BD Phoenix, MicroScan) frequently misidentify *C. auris* as *Candida haemulonii*, *Candida duobushaemulonii*, *Candida sake*, *Candida intermedia*, or *Candida parapsilosis* (5). Accordingly, the US Centers for Disease Control and Prevention (CDC) recommends confirmatory testing using more specific identification methods when these species are reported (5).

MALDI-TOF MS provides reliable identification of *C. auris* when updated databases are used, including the Bruker MALDI Biotyper CA System (Version Claim 4) or bioMérieux VITEK MS IVD v3.2 (5). Molecular assays, such as nucleic acid amplification tests (NAATs) and sequencing targeting the 28S rDNA or Internal Transcribed Spacer (ITS) region, also enable accurate species identification and are increasingly implemented in clinical and public health laboratories (5–8). Although many NAATs are laboratory-developed tests and therefore not cleared by the FDA, one FDA-cleared assay is currently available: the Diasorin Simplexa C. auris Direct. Both culture-based and molecular approaches are CDC-endorsed, with molecular assays offering faster turnaround times but generally at higher costs (9, 10).

Antifungal susceptibility testing is critical because *C. auris* frequently demonstrates resistance to multiple antifungal classes. The Clinical and Laboratory Standards Institute (CLSI) and the European Committee on Antimicrobial Susceptibility Testing (EUCAST) have both developed reference methods for antifungal susceptibility testing based on broth microdilution (11). Although CLSI has not established definitive breakpoints for *C. auris*, epidemiological cutoff values (ECVs) are available for selected agents, including echinocandins such as micafungin and caspofungin. Because MIC distributions vary considerably by clade and region (1), many laboratories using CLSI-based methods, including Sensititre YeastOne assays, continue to rely on the CDC tentative breakpoints to guide interpretation (12). In contrast, EUCAST provides ECVs and clinical breakpoints derived from large global isolate collections (13).

*C. auris* generally exhibits higher antifungal resistance rates than most *Candida* species (1, 14, 15). Fluconazole resistance is often linked to *ERG11* mutations encoding altered lanosterol 14α-demethylase, while echinocandin resistance is typically associated with *FKS1* mutations affecting the β−1,3-d-glucan synthase complex (16). Pan-resistant isolates against triazoles, polyenes, and echinocandins have also been described, underscoring the importance of surveillance and stewardship (14). Local *C. auris* isolates at our institution demonstrate high fluconazole resistance, followed by amphotericin B resistance and variable echinocandin MICs, consistent with national trends (17).

Current guidelines recommend treating only clinical infection, not asymptomatic colonization (18). Echinocandins remain first-line therapy for most invasive *C. auris* infections, while amphotericin B deoxycholate is preferred for infants under 2 months due to its high efficacy, broad spectrum activity, and relatively low resistance risk (18, 19). Close clinical and microbiologic monitoring is essential because resistance may emerge during therapy. Step-down to azole therapy is appropriate only after clinical improvement and culture clearance. For CNS candidiasis specifically, liposomal amphotericin B with or without flucytosine, or amphotericin B deoxycholate with or without flucytosine, is recommended. Echinocandins are not preferred due to poor CNS penetration (19). In this case, high-dose micafungin was temporarily administered (200 mg daily rather than the standard 100 mg) to augment potential CNS exposure while awaiting amphotericin B susceptibility results. When considering long-term antifungal suppression therapy, factors such as the presence of indwelling hardware, evidence of colonization or persistent infection, multidrug-resistant isolates, and the limited CNS penetration of available antifungal drugs should be taken into account.

For multidrug-resistant isolates, investigational antifungals such as Ibrexafungerp (NCT03363841: Phase III trial Completed) and fosmanogepix (NCT05321858: Ongoing Phase III trial) offer promising options. Ibrexafungerp is a first-in-class triterpenoid glucan synthase inhibitor with activity against *C. auris,* and CDC lists it as a potential choice for pan-resistant *C. auris* (15). Fosmanogepix inhibits Gwt1, a fungal-specific inositol acyltransferase essential for cell wall assembly, and demonstrates potent activity against resistant *Candida* species, including *C. auris,* with favorable pharmacokinetics and oral bioavailability (20). While no commercial susceptibility testing panels currently include ibrexafungerp or fosmanogepix, the Fungus Testing Laboratory at UT Health San Antonio offers commercial ibrexafungerp susceptibility testing for yeasts and molds. In contrast, commercial susceptibility testing for fosmanogepix is not currently available.

In summary, this case illustrates the substantial challenges posed by *C. auris*, especially in CNS infections where therapeutic options remain limited. The patient’s prolonged course, multiple antifungal regimen adjustments, and persistent culture positivity despite aggressive treatment highlight the organism’s resilience and multidrug-resistant potential. Prompt species identification and timely susceptibility testing were essential for guiding management, and emerging agents such as fosmanogepix may offer future therapeutic options. Continued vigilance, robust infection prevention, and development of novel antifungal agents remain critical as *C. auris* continues to spread globally.

## Data Availability

All data analyzed and presented in this case report are available from the corresponding author on reasonable request.
